# Meta-analysis of the relationship between interleukin-6 levels and the prognosis and severity of acute coronary syndrome

**DOI:** 10.6061/clinics/2021/e2690

**Published:** 2021-06-29

**Authors:** Chongzhe Yang, Zhiyong Deng, Jie Li, Zhilei Ren, Feng Liu

**Affiliations:** Department of Geriatrics, National Key Clinic Specialty, Guangzhou First People's Hospital, School of Medicine, South China University of Technology, Guangzhou, Guangdong 510180, China.

**Keywords:** Acute Coronary Syndrome, Interleukin 6, Major Adverse Cardiovascular Event, Meta-Analysis

## Abstract

This study aimed to explore the relationship between plasma interleukin 6 (IL-6) levels, adverse cardiovascular events, and the severity of acute coronary syndrome (ACS). A literature review was performed of studies regarding IL-6 and ACS extracted from databases including EMBASE, Cqvip, MEDLINE, Web of Knowledge, PubMed, Cochrane Library, China National Knowledge Infrastructure, and Wanfang data. The Newcastle-Ottawa scale (NOS) was used to evaluate the quality of the literature. The literature was screened, its quality was evaluated, and relevant data were extracted for performing meta-analysis using RevMan software (version 5.3). A total of 524 studies were included in the initial survey. After several rounds of screening and analysis, six studies met the inclusion criteria and underwent meta-analysis using a fixed-effect model. Patients were divided into non-severe and severe groups based on the concentration of high-sensitivity C-reactive protein. Meta-analysis of the relationship between IL-6 and the severity of ACS showed that the plasma IL-6 level of patients in the severe group was significantly higher than that of patients in the non-severe group (*p*<0.00001). Additionally, patients with experience of major adverse cardiovascular events had significantly higher plasma IL-6 levels than did patients without experience of such events (*p*<0.00001). In summary, patients with ACS and high IL-6 levels tended to be in a critical condition, with a higher risk of adverse cardiovascular events and worse prognosis. Thus, IL-6 levels could indicate whether patients with ACS may have adverse cardiovascular events and determine the severity of ACS.

## INTRODUCTION

Several cytokines (tumor necrosis factors alpha [TNF-α], interstitial interleukins 1 [IL-1], chemokines, adipokines, and interferons) are involved in the occurrence of acute coronary syndrome (ACS). The interaction of these cytokines is involved in the process of atherosclerotic plaque destabilization, which results in ACS. TNF-α and IL-1 families are especially involved in the accumulation of inflammatory cells, platelet aggregation that accelerates the formation of unstable plaques, myocardial cell apoptosis, and ventricular remodeling following myocardial infarction.

Cytokines such as interleukin 6 (IL-6) have noticeable prognostic value in patients with ACS (1). Since its detection is rapid and easy to perform, IL-6 has gradually become a commonly used indicator in clinical practice (2) and is strongly associated with the severity of ACS. Plasma IL-6 levels are significantly elevated in patients with coronary heart disease and are not affected by other risk factors (3). Particularly in patients with unstable angina (UA), plasma IL-6 levels are three times higher than those of healthy controls and are closely related to the concentration of high-sensitivity C-reactive protein (hs-CRP), indicating that IL-6 may be involved in the early acute-phase reaction of ACS (4). Studies have further found that patients with long-term acute myocardial infarction (AMI) have significantly higher plasma IL-6 levels than do healthy people, and their IL-6 levels are independent of risk factors such as blood pressure, diabetes, and total cholesterol. Some studies have microscopically explored the mechanism by which IL-6 promotes ACS, proving that IL-6 promotes macrophage activation, infiltration, and increased expression of low-density lipoprotein (LDL) receptors, thereby enhancing macrophage uptake of LDL and inducing increased expression of tissue factor. These mechanisms contribute to the inflammatory process of atherosclerosis (5). As a sensitive indicator of inflammation, hs-CRP is the most commonly used biomarker for coronary heart disease. IL-6 is also an inflammatory marker of local coronary plaques and peripheral blood circulation. It can promote the development of coronary heart disease by causing plaque instability and rupture. Wang et al. (6) confirmed that serum hs-CRP and IL-6 levels can be used to determine plaque stability, which is closely related to ACS. Therefore, IL-6 levels are valuable for predicting whether a patient with ACS will experience adverse cardiovascular events.

Many studies have focused on the connections between IL-6 and cardiovascular disease severity, as well as the long-term prognosis of ACS (7). Elevated levels of hs-CRP and IL-6 are vital and independent markers of poor prognosis and high mortality in patients with ACS (8); however, studies with large sample sizes are lacking. Therefore, this study discusses the relationship between IL-6, ACS severity, and the incidence of major adverse cardiovascular events (MACE) using a meta-analysis.

## MATERIALS AND METHODS

The inclusion criteria were as follows: (1) full-text studies published in Mandarin or English languages; (2) studies exploring the relationship between IL-6 and the severity of ACS or MACE; (3) studies on the prognosis of ACS and occurrence of MACE as outcomes; and (4) studies in which the severity of ACS was evaluated by measuring the level of hs-CRP such that it is convenient to integrate the data.

The exclusion criteria were as follows: (1) reviews, case reports, or non-clinical studies; (2) studies with insufficient original data or incomplete data; (3) studies that failed to clearly diagnose the study subject; (4) studies that did not exclude other risk factors affecting IL-6 levels. This article does not contain any studies with human participants performed by any of the authors.

### Literature search and screening

#### Search method

Databases including PubMed, Cqvip, MEDLINE, EMBASE, China National Knowledge Infrastructure, and Wanfang were explored. The deadline for the literature to be included was May 31, 2020. English databases were searched using a combination of the following search terms: ((Acute Coronary Syndromes[major]) OR (Coronary Syndrome, Acute) OR (Coronary Syndromes, Acute) OR (Syndrome, Acute Coronary) OR (Syndromes, Acute Coronary)) AND ((Interleukin 6) OR (IL6) OR (B-Cell Stimulatory Factor 2) OR (B-Cell Stimulatory Factor-2) OR (Differentiation Factor-2, B-Cell) OR (Differentiation Factor 2, B Cell) OR (B Cell Differentiation Factor 2) OR (B-Cell Differentiation Factor-2) OR (BSF-2) OR (Hybridoma Growth Factor) OR (Growth Factor, Hybridoma) OR (IFN-beta 2) OR (Plasmacytoma Growth Factor) OR (Growth Factor, Plasmacytoma) OR (Hepatocyte-Stimulating Factor) OR (Hepatocyte-Stimulating Factor) OR (MGI-2) OR (Myeloid Differentiation-Inducing Protein) OR (Differentiation-Inducing Protein, Myeloid) OR (Myeloid Differentiation-Inducing Protein) OR (B-Cell Differentiation Factor) OR (B Cell Differentiation Factor) OR (Differentiation Factor, B-Cell) OR (Differentiation Factor, B Cell) OR (IL-6) OR (Interferon beta-2) OR (Interferon beta 2) OR (beta-2, Interferon) OR (B Cell Stimulatory Factor-2) OR (B Cell Stimulatory Factor 2)). When searching Chinese databases, the following titles or keywords were used: “acute coronary syndrome” OR “ACS” AND “interleukin 6” OR “IL-6” in Mandarin.

### Data extraction

The title, first author, time of publication, type of study, quantitative data of IL-6, type of ACS diagnosed, MACE, and sample size were extracted from the literature search.

### Quality evaluation

Two researchers conducted the literature search, read the full text, and screened the literature according to the inclusion and exclusion criteria. In case a conflict of opinion arose, a third researcher joined the discussion, and the result was decided through consensus. The quality of the selected studies was evaluated using the Newcastle-Ottawa Scale (NOS). The NOS consists of eight items with three subscales, and the total maximum score of the three subscales is 9. A study that scored ≥7 was arbitrarily considered to be of high quality since a standard criterion for what constitutes a high quality study has not yet been universally established.

### Statistical analyses

RevMan software (version 5.3) (The Cochrane Collaboration, Copenhagen, Denmark) was used for performing statistical analyses. The I^2^ test was used to determine heterogeneity. If I^2^ was <50%, the studies were considered homogeneous, following which the fixed-effect model was used to analyze the included data; if I^2^ was ≥50%, it was considered that there was heterogeneity among the various studies, and the random effects model was adopted. In the study of the relationship between IL-6 and the severity and prognosis of ACS, IL-6 levels were quantified and analyzed using weighted standard deviation (SD). In the meta-analysis, *p*<0.05, indicated that the difference was statistically significant. A funnel chart was constructed to perform a bias analysis of the selected studies, and the results were represented using forest plots.

## RESULTS

### Basic data of the included literature

**Figure 1:**
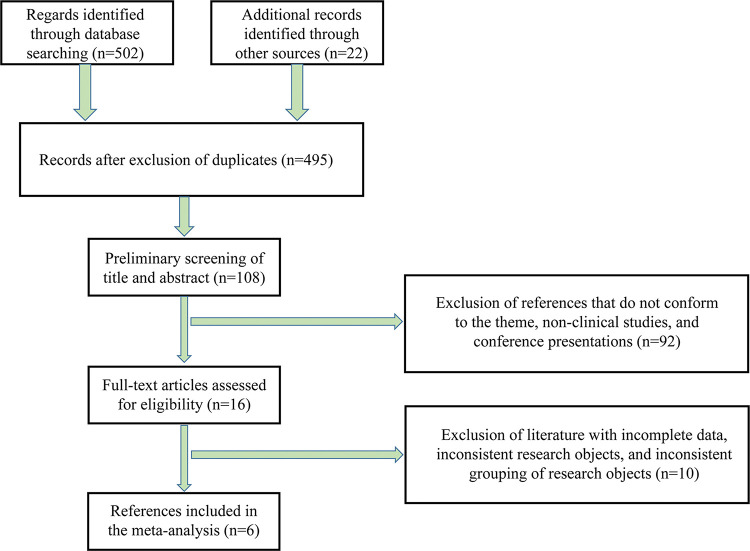
Process of document inclusion.

A preliminary search of the literature was conducted, and 524 studies were obtained. After excluding duplicate studies, 495 studies remained. After reading the abstract and title, 108 studies were retained. The exclusion criteria were used to screen the 108 studies, following which the quality of the studies was evaluated, and six studies were finally selected (6,9-13) (Figure 1). Among them, three studies analyzed the relationship between IL-6 (measured by ELISA) and the severity of ACS, whereas the remaining three explored the relationship between IL-6 and the prognosis of ACS. All six studies scored 7-9 points on the NOS, indicating high quality (Table 1).

### Relationship between IL-6 and the severity and prognosis of ACS

The patients were divided into those who experienced MACE during hospitalization and long-term follow-up (with event) and those who did not (without event). Thereafter, the specific values of IL-6 and interquartile range (IQR) or SD for each group were extracted (Table 2). Data regarding the relationship between plasma IL-6 levels and the severity of ACS were extracted, including grouping and number of patients in each group and mean values as well as IQRs of hs-CRP and IL-6 levels (Table 3). For the meta-analysis, ST-elevation myocardial infarction (STEMI), non-ST-elevation myocardial infarction (NSTEMI), and UA were grouped according to hs-CRP levels. The group with lower hs-CRP was regarded as the “non-severe group”, and the group with higher hs-CRP as the “severe group.”

### Meta-analysis of the relationship between IL-6 and the severity of ACS

**Figure 2:**
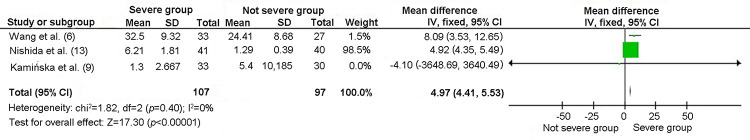
Forest plot of the relationship between interleukin-6 and the severity of acute coronary syndrome. CI, confidence interval; df, degrees of freedom; SD, standard deviation.

Three of the selected studies analyzed the relationship between IL-6 levels and the severity of ACS. A heterogeneity test was conducted on the selected studies. These studies showed appropriate homogeneity [chi^2^=1.82, degree of freedom (df)=2, *p*=0.40, I^2^=0%]; thus, the fixed-effect model was used. The results of the meta-analysis showed that patients in the severe group had increased IL-6 levels than did patients in the non-severe group (mean difference [MD]=4.97, 95% confidence interval [CI]=4.41 to 5.53, *p*<0.00001) (Figure 2).

### Meta-analysis of the relationship between IL-6 and MACE in ACS

**Figure 3:**
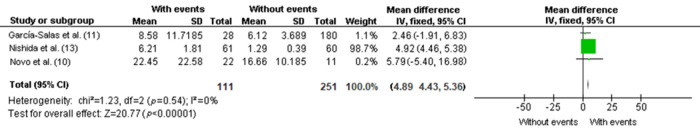
Forest plot of the relationship between interleukin-6 and the risk of major adverse cardiovascular events during acute coronary syndrome follow-up. CI, confidence interval; df, degrees of freedom; SD, standard deviation.

Three of the selected studies analyzed the relationship between IL-6 and MACE in ACS. The heterogeneity test revealed appropriate homogeneity among these studies (chi^2^=1.23, df=2, *p*=0.54, I^2^=0%). Therefore, a fixed-effect model was used for the analysis. Meta-analysis revealed that patients in the group with poor prognosis (with event) showed increased IL-6 levels than did patients in the group with favorable prognosis (without event) (MD=4.89, 95% CI=4.43 to 5.36, *p*<0.00001) (Figure 3).

## DISCUSSION

Various theories, including platelet formation, injury response, lipid plaque formation, and inflammation, are used to explain the pathophysiology of coronary heart disease. Among these, the inflammation theory has received emerging interest in recent years. Plasma concentrations of IL-6 and hs-CRP (inflammation markers) have predictive value for the prognosis of patients with myocardial infarction. Plasma IL-6 levels are correlated with plasma hs-CRP concentrations, and there is a positive correlation between the former and creatine kinase-myocardial band levels (14). IL-6 is also related to the underlying symptoms of early ACS and is conducive to confirming the diagnosis (15). Exploring the role of IL-6 in the severity of ACS and occurrence of MACE can not only deepen the understanding of the inflammation theory (9) but also link the inflammation theory with other well-studied theories such as the lipid formation theory (16). Early diagnosis of ACS is essential to delay the development of the disease. The diagnostic criteria used in our meta-analysis not only identified the type of ACS, but also its severity and prognosis. Therefore, our results contribute to the present literature by quantifying the relationship between IL-6 levels and the prognosis and severity of ACS.

**Figure 4:**
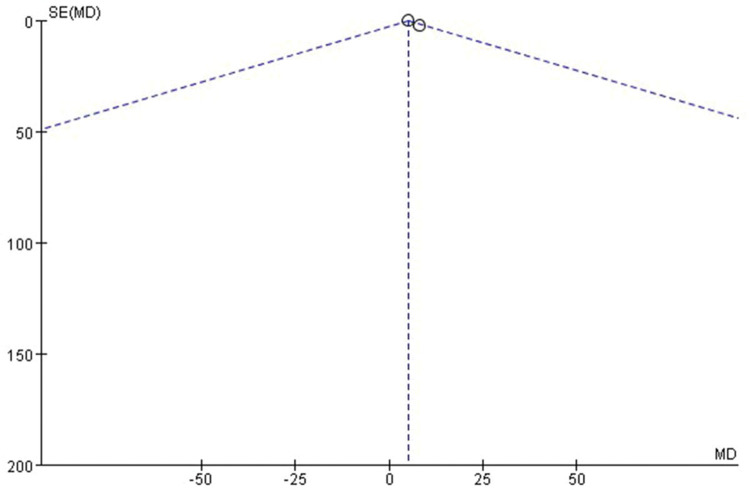
Funnel chart of the relationship between interleukin-6 and the severity of acute coronary syndrome. SE, standard error; MD, mean difference.

**Figure 5:**
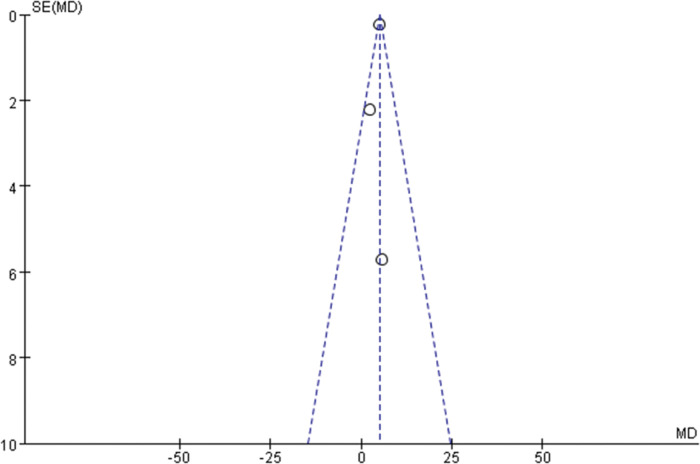
Funnel chart of the relationship between interleukin-6 and the risk of major adverse cardiovascular events during acute coronary syndrome follow-up. SE, standard error; MD, mean difference.

This meta-analysis included six studies published from 2011 to 2018, of which three studies were prospective and three were retrospective. The I^2^ values of all studies were <50%; thus, the fixed-effect model was used for the meta-analysis. The forest plot shows that the plasma IL-6 levels of patients in the severe group were significantly higher than those of patients in the non-severe group (*p*<0.00001). The meta-analysis of the relationship between IL-6 and the prognosis of ACS showed that patients with MACE had significantly higher IL-6 levels than did those without MACE (*p*<0.00001) (Figures 2 and 3). Our findings shows that patients with ACS and higher IL-6 levels tended to be in a critical condition and had worse prognosis than did patients with ACS but lower IL-6 levels. Therefore, we inferred that plasma IL-6 levels were indicative of the severity and prognosis of patients with ACS. Due to the limited number of studies included, there was a risk of potential publication bias. The bias analysis was conducted using RevMan statistical software, and no publication bias was found, indicating that the results of this study are relatively stable and reliable (Figures 4 and 5).

Coronary heart disease can be classified as stable angina (SA), UA, NSTEMI, and STEMI, of which the latter three are collectively referred to as ACS, while the last two are collectively referred to as AMI. Of the three studies on the relationship between IL-6 and ACS severity, Wang et al. (6) divided patients with ACS into those with AMI and UA, whereas the other two studies categorized ACS patients into those with UA, NSTEMI, and STEMI with higher specificity. The grouping method differed between studies but did not affect the discussion of the results.

Patients with stable angina have significantly higher levels of cytokines, such as IL-6 and TNF-α, than do patients with normal coronary arteries (17,18). Moreover, IL-6 levels are significantly higher in patients with ACS than in patients with stable angina (19). These findings prove that changes in IL-6 concentration may reflect the degree of inflammation in coronary heart disease and the severity of coronary atherosclerosis. UA, NSTEMI, and STEMI were qualitatively distinguished based on the characteristics of chest pain, electrocardiogram, and the concentration of myocardial necrosis markers. From a micropathological perspective (20,21), the three types of coronary artery stenosis ranged from incomplete infarction to complete infarction. The degree of infarction can be assessed based on invasive coronary angiography or non-invasive computed tomography angiography (CTA), the accuracy of which is affected by vascular calcification. In addition, intravascular ultrasound (IVUS) or optical coherence tomography (OCT) can be used to determine the size and stability of the plaque to comprehensively assess the severity of ACS. However, these tests are expensive, complex, and lack a quantitative assessment of the prognosis and severity of ACS in patients. The amount of IL-6 and other inflammatory factors in plasma, as well as the hs-CRP concentration, provide opportunities for the quantitative analysis of the prognosis and severity of ACS in patients (22). There is also a close relationship between inflammatory indicators; for example, a positive correlation exists between serum hs-CRP and IL-6 levels (6). Both serum hs-CRP and IL-6 levels can determine the stability of plaques, with greater instability of the plaques indicating a more critical ACS. Moreover, increased inflammatory activity is noted in patients with ACS. The levels of several inflammatory cytokines are increased in patients with ACS (23). Therefore, IL-6 levels can be an alternative/adjunct to cumbersome and expensive techniques, such as digital subtraction angiography, CTA, IVUS, and OCT, to predict and evaluate the severity of ACS. Several studies have shown that serum hs-CRP and IL-6 levels can be used as indicators to assess the degree of coronary heart disease (22) and may also be used to reflect the activity of plaque in patients with coronary heart disease; therefore, these biomarkers are of great significance for the clinical diagnosis and risk assessment of patients with ACS (24).

The merit of this study is that we included relevant worldwide studies and conducted a meta-analysis to expand the sample size and increase the accuracy of the results. The results of our meta-analysis indicate that higher IL-6 values reflect ACS patients with more critical conditions, worse prognosis, and higher probability of MACE. Compared with that of the study by Li et al. (25), the included literature in our paper involved more people and covered multiple countries, making the content of the discussion more generalizable and applicable. Our findings hold significance because we subdivided ACS into three types, namely NSTEMI, STEMI, and UA, allowing us to discuss the severity of ACS in detail. However, there remain few limitations associated with our meta-analysis. First, due to the inconsistency of the classification methods, the three different disease subtypes of ACS could not be separately analyzed using the meta-analysis. Liu et al. (12) used the number of diseased vessels for risk stratification, which is inconsistent with other studies. Second, the grouping based on severity was complicated because none of the selected studies mentioned the SYNTAX, GRACE, and Gensini scores, and the relationship between these scores is yet to be determined. Finally, when evaluating whether IL-6 affects the occurrence of MACE, there was no data regarding the incidence of MACE, and patients were crudely classified into those who did/did not have MACE. In addition, the classification and definition of MACE differed across the studies. Therefore, the relationship between IL-6 and the occurrence of ACS requires further investigation. Furthermore, data heterogeneity regarding sex, age, ethnicity, and other factors also limits the study’s findings.

In summary, patients with ACS and high IL-6 levels experience severe changes in their condition and worse prognosis. Therefore, plasma IL-6 levels may indicate the severity of ACS and occurrence of MACE.

## AUTHOR CONTRIBUTIONS

Yang C and Deng Z designed the experiments and wrote the manuscript. Li J and Ren Z carried out experiments and analyzed the experimental results. Liu F revised the manuscript. All authors approved the final version of the manuscript.

## Figures and Tables

**Table 1:** Basic information about the selected studies.

Author (s)	Population (ethnicity)	Published time	Research type	Patients’ diagnosis	Number of patients	Severity degree	Prognosis	NOS score
Kamińska et al. (9)	Poland	2018	Retrospective	ACS	93	hs-CRP	NA	9
Novo et al. (10)	Italy	2015	Prospective	STEMI	33	NA	MACE	8
Wang et al. (6)	China	2014	Retrospective	ACS	60	hs-CRP	NA	8
García-Salas et al. (11)	Spain	2014	Prospective	NSTEMI-ACS and troponin- negative low- risk patients	212	NA	MACE	7
Liu et al. (12)	China	2013	Retrospective	ACS	40	Number of diseased vessels	NA	9
Nishida et al. (13)	Japan	2011	Prospective	ACS	121	hs-CRP	MACE	7

AMI, acute myocardial infarction; STEMI, ST-elevation myocardial infarction; NSTEMI, non-ST-elevation myocardial infarction; ACS, acute coronary syndrome; hs-CRP, high-sensitivity C-reactive protein; NA, not available; NOS, Newcastle-Ottawa Scale.

**Table 2:** Relationship between interleukin-6 and the prognosis of acute coronary syndrome.

Author (s)	Population (ethnicity)	Follow-up time	With event		Without event	
			Median IL-6 level (IQR), pg/mL	Total	Median IL-6 level (IQR), pg/mL	Total
Novo et al. (10)	Italy	During hospitalization and in a 6 year long-term follow-up period	22.45 (16.05-46.54)	22	16.66 (15.25-29.00)	11
García-Salas et al. (11)	Spain	6 months	8.58 (5.13-20.95)	28	6.12 (4.16-9.14)	180
Nishida et al. (13)	Japan	34.4 months	6.21±1.81*	61	1.29±0.39	60

IL-6, interleukin-6; IQR: interquartile range; parentheses and ± indicate IQR and standard deviation (SD), respectively. * denotes *p*<0.01. Literature review results on the prognosis of IL-6 and acute coronary syndrome (ACS) lesions in the included studies were extracted. Based on the occurrence of major adverse cardiovascular events (MACE) during hospitalization and long-term follow-up, patients were divided into two groups: those who experienced MACE (with events) and those who did not (without events). The specific value of IL-6 in each group, IQR or SD, as well as the grouping and the number of patients in each group were extracted.

**Table 3:** Relationship between interleukin-6 and the severity of acute coronary syndrome.

Author (s)	Population (ethnicity)	Published time	Groups	Groups of cases	Severity degree				IL-6, pg/mL			
Nishida et al. (13)	Japan	2011	STEMI/NSTEMI/UA	41/40/40		STEMI	NSTEMI	UA	STEMI		NSTEMI	UA
					hs-CRP (mg/L)	4.7±5.9	1.6±1.8	1.2±2.3	6.21±1.81		2.62±0.56	1.29±0.39
Kamińska et al. (9)	Poland	2018	STEMI/NSTEMI/UA	33/30/30		STEMI	NSTEMI	UA	STEMI		NSTEMI	UA
					hs-CRP (mg/L)	3.5 (1.4-5.0)	2.0 (0.9-3.9)	1.3 (0.9-2.9)	12.8 (9.9-17.4)		18.8 (12.2-24.1)	5.4 (2.2-11.0)
Wang et al. (6)	China	2014	AMI/UA	33/27		AMI	UA		AMI			UA
					hs-CRP (mg/L)	8.91±2.12	5.95±1.60		32.50±9.32			24.41±8.68

AMI, acute myocardial infarction; IL-6, interleukin-6; STEMI, ST-elevation myocardial infarction; NSTEMI, non-ST-elevation myocardial infarction; ACS, acute coronary syndrome; UA, unstable angina; hs-CRP, high-sensitivity C-reactive protein. Data are shown as the mean±standard deviation; * *versus* control group, *p*<0.01; AMI includes STEMI and NSTEMI. Data regarding grouping, number of patients in each group, hs-CRP, standard deviation, and interquartile range of IL-6 were extracted from the included studies that mentioned the association between plasma IL-6 size and the severity of ACS.
